# Ketogenic β‐hydroxybutyrate regulates β‐hydroxybutyrylation of TCA cycle‐associated enzymes and attenuates disease‐associated pathologies in Alzheimer's mice

**DOI:** 10.1111/acel.14368

**Published:** 2024-10-16

**Authors:** Wanhong Han, Bingchang Zhang, Wenpeng Zhao, Wentao Zhao, Jiawei He, Xiansheng Qiu, Liang Zhang, Xiuyan Wang, Yong Wang, Hanwen Lu, Yaya Zhang, Yuanyuan Xie, Yanyan Geng, Wujie Zhao, Qionghui Huang, Yun‐wu Zhang, Zhanxiang Wang

**Affiliations:** ^1^ Department of Neurosurgery and Department of Neuroscience, Fujian Key Laboratory of Brain Tumors Diagnosis and Precision Treatment, Xiamen Key Laboratory of Brain Center, the First Affiliated Hospital of Xiamen University, School of Medicine Xiamen University Xiamen China; ^2^ Xiamen Key Laboratory of Brain Center, the First Affiliated Hospital of Xiamen University, and Fujian Provincial Key Laboratory of Neurodegenerative Disease and Aging Research, Institute of Neuroscience, School of Medicine Xiamen University Xiamen Fujian China; ^3^ Department of Medical Oncology The First Affiliated Hospital of Xiamen University Xiamen China; ^4^ Department of Anesthesiology The First Affiliated Hospital of Xiamen University Xiamen China

**Keywords:** Alzheimer's disease, β‐Hydroxybutyrylation, citrate synthase, ketogenic diet, succinate‐CoA ligase subunit alpha, TCA cycle

## Abstract

Lysine β‐hydroxybutyrylation (Kbhb) is a post‐translational modification that has recently been found to regulate protein functions. However, whether and how protein Kbhb modification participates in Alzheimer's disease (AD) remains unknown. Herein, we carried out 4D label‐free β‐hydroxybutylation quantitative proteomics using brain samples of 8‐month‐old and 2‐month‐old APP/PS1 AD model mice and wild‐type (WT) controls. We identified a series of tricarboxylic acid (TCA) cycle‐associated enzymes including citrate synthase (CS) and succinate‐CoA ligase subunit alpha (SUCLG1), whose Kbhb modifications were decreased in APP/PS1 mice at pathological stages. Sodium β‐hydroxybutyrate (Na‐β‐OHB) treatment markedly increased Kbhb modifications of CS and SUCLG1 and their enzymatic activities, leading to elevated ATP production. We further found that Kbhb modifications at lysine 393 site in CS and at lysine 81 site in SUCLG1 were crucial for their enzymatic activities. Finally, we found that β‐OHB levels were decreased in the brain of APP/PS1 mice at pathological stages. While ketogenic diet not only significantly increased β‐OHB levels, Kbhb modifications and enzymatic activities of CS and SUCLG1, and ATP production, but also dramatically attenuated β‐amyloid plaque pathologies and microgliosis in APP/PS1 mice. Together, our findings indicate the importance of protein Kbhb modification for maintaining normal TCA cycle and ATP production and provide a novel molecular mechanism underlying the beneficial effects of ketogenic diet on energy metabolism and AD intervention.

## Graphical abstract

We demonstrate that β‐hydroxybutyrate (BHB) induces β‐hydroxybutyrylation (Kbhb) of citrate synthase (CS) and succinyl‐CoA ligase (SUCLG1), both are enzymes in the tricarboxylic acid (TCA) cycle. The Kbhb of CS and SUCLG1 enhances ATP production, contributing to the attenuation of AD‐associated pathologies in APP/PS1 mice by ketogenic diet treatment.

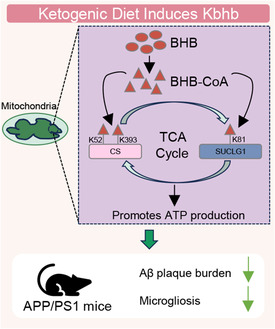

**Abbreviations**
AD: Alzheimer's disease
CS: citrate synthase
DLD: dihydrolipoyl dehydrogenase
ETC: electron transport chain
IDH: isocitrate dehydrogenase
Kbhb: lysine β‐hydroxybutylation
KD: ketogenic diet
MDH2: malate dehydrogenase 2
SDH: succinate dehydrogenase
SUCLG1: succinate‐CoA ligase subunit alpha
TCA: tricarboxylic acid
WT: wild‐type
β‐OHB: β‐hydroxybutyrate

## 1 INTRODUCTION

Alzheimer's disease (AD) is a neurodegenerative disease and a leading cause of dementia (Knopman et al., 2021). As the population ages, the prevalence of AD continues to increase (Ballard et al., 2020). Individuals with AD suffer from cognitive memory impairment, which hinders their ability to live independently (Murphy, 2019). Despite notable progress in comprehending the pathogenesis of AD, a cure remains elusive. Hence, it is imperative to conduct comprehensive research into the pathological mechanisms of AD.

As a highly ATP‐demanding tissue, the brain primarily relies on glucose metabolism to obtain energy and ensure the normal functioning of the nervous system. However, during AD occurrence, multiple pathogenic factors lead to glucose metabolism dysfunction in the brain of patients (Butterfield & Halliwell, 2019). Glucose hypometabolism is widely recognized as one important contributing factor in AD (Kuehn, 2020). Molecular mechanism studies have identified impairments in glucose consumption, glycolysis, the tricarboxylic acid (TCA) cycle, and the electron transport chain (ETC) metabolic pathways in the mitochondria in AD patients. Brain glucose hypometabolism leads to energy deficiency in AD. Since ketone bodies serve as an alternative energy source for the brain upon glucose deficiency, ketogenic intervention may become a potential energy rescue approach for AD (Cunnane et al., 2020; Henderson, 2008). Indeed, several studies have shown the effectiveness of ketogenic diet (KD) in alleviating symptoms of AD (Avgerinos et al., 2020; Henderson et al., 2009; Taylor et al., 2018; Xu et al., 2020).

β‐hydroxybutyrate (β‐OHB) is a main product of ketogenesis (Newman & Verdin, 2014). Upon glucose hypometabolism, β‐OHB is subjected to ketolysis and converted into acetyl‐CoA, which enters the TCA cycle for ATP production. β‐OHB has been found to enhance cognition and inhibit Aβ plaque deposition, microglial cell proliferation, and neuroinflammation in AD mouse models through promoting mitochondrial metabolism, regulating signaling molecules, increasing histone acetylation, and affecting the clearance of Aβ and Tau proteins (Shippy et al., 2020; Wang et al., 2022; Wu et al., 2020). Recently, a role of β‐OHB in regulating protein function through lysine β‐hydroxybutylation (Kbhb) modification has been identified. For example, β‐OHB‐induced histone Kbhb modification is an epigenetic modification that regulates gene expression, and H3K9 Kbhb modification is important for CD8^+^ T‐cell memory development (Xie et al., 2016; Zhang et al., 2020); AHCY Kbhb modification regulates liver metabolism (Koronowski et al., 2021); p53 Kbhb modification contributes to the reduction of cell growth arrest and apoptosis (Liu et al., 2019); and CaMKII‐α Kbhb modification alleviates the reinstatement of cocaine‐conditioned place (Li et al., 2022). However, to our knowledge, so far there is no report regarding the involvement of protein Kbhb modification in the onset and progression of AD.

In this study, we aimed to identify AD‐associated protein Kbhb modifications using 4D label‐free β‐hydroxybutylation quantitative proteomics. We found that Kbhb modifications of citrate synthase (CS) and succinate‐CoA ligase subunit alpha (SUCLG1), both of which are key enzymes in the TCA cycle, were significantly decreased in the brain of APP/PS1 mice at pathological stages. Both Na‐β‐OHB treatment in vitro and ketogenic diet in vivo promoted CS and SUCLG1 Kbhb modifications and their enzymatic activities, leading to elevated ATP production. Ketogenic diet further attenuated β‐amyloid plaque pathologies and microglial overactivation in the brain of APP/PS1 mice.

## 2 MATERIALS AND METHODS

### 2.1 Animals

Male APP/PS1 mice (B6C3‐Tg (APPswe, PSEN1dE9) 85Dbo/Mmjax) and their wild‐type (WT) littermates were purchased from Gene&Peace Biotech Co, Ltd. (Jiangsu, China). Mice were housed in the animal room under a standard 12‐h light/12‐h dark cycle with a constant temperature. All animal experiments were conducted in accordance with the guidelines and regulations approved by the Animal Care and Use Committee of Xiamen University (XMULAC20230234).

### 2.2 Cell and cell culture

293T cells and HT22 cells were cultured in Dulbecco's Modified Eagle's Medium (SH30243.01, HyClone, USA) containing 10% fetal bovine serum (WS500T, ABW, China) and 1% penicillin/streptomycin (15140‐122, Gibco, USA) and grown in 5% CO_2_ at 37°C.

### 2.3 Mouse brain β‐hydroxybutyrylation proteomics analysis

Brain tissues from 2‐month‐old and 8‐month‐old APP/PS1 mice and respective WT controls were subjected to β‐hydroxybutyrylation proteomics analysis, which was performed by PTM Biolabs Inc. (Hangzhou, China). Detailed procedures are described as the following:

#### 2.3.1 Protein extraction

Half of the mouse brain tissues were lysed by four volumes of lysis buffer (8 M urea, 1% Protease Inhibitor Cocktail) and sonicated 3 times on ice with a high‐intensity ultrasonic processor. Debris were removed by centrifugation at 12,000 ***g*** at 4°C for 10 min. The supernatant was collected, and the protein concentration was determined with a BCA kit.

#### 2.3.2 Trypsin digestion

Collected protein solutions were reduced with 10 mM dithiothreitol for 1 h at 37°C and alkylated with 20 mM iodoacetamide for 45 min at room temperature in darkness. The excess iodoacetamide was blocked by 20 mM cysteine. Protein solutions were diluted with 100 mM NH_4_HCO_3_ to reduce the urea concentration to 1 M. Trypsin was added at 1:50 trypsin‐to‐protein mass ratio for the first digestion overnight and at 1:100 trypsin‐to‐protein mass ratio for a second 4 h digestion. 18 mg of digested proteins were used for subsequent experiments.

#### 2.3.3 Peptide fractionation and immunoaffinity enrichment

Peptides were fractionated into 23 fractions by high pH reverse‐phase HPLC with an Agilent 300 Extend C18 column (5 μm particles, 4.6 mm ID, 250 mm length) and dried by vacuum centrifugation. To enrich Kbhb‐modified peptides, peptides dissolved in NETN buffer (100 mM NaCl, 1 mM EDTA, 50 mM Tris–HCl, 0.5% NP‐40, pH 8.0) were incubated with 30 μL prewashed anti‐pan‐Kbhb antibody beads (PTM Biolabs Inc) at 4°C overnight with gentle shaking. The beads were washed for four times with NETN buffer and twice with ddH_2_O. The bound peptides were eluted from the beads with 0.1% trifluoroacetic acid. Eluted peptides were vacuum‐dried and subjected to LC–MS/MS.

#### 2.3.4 LC–MS/MS analysis

Peptides were dissolved in 0.1% formic acid and loaded onto reversed‐phase analytical column (10 cm length with 75 μm inner diameter) packed with Reprosil 100 C18 resin (3 μm particle size, Dr. Maisch GmbH, Ammerbuch, Germany). The separation of peptides was carried out using a gradient of 5%–80% HPLC buffer B (0.1% formic acid in 90% acetonitrile, v/v) in buffer A (0.1% formic acid in water, v/v) at a flow rate of 300 nL/min over 60 min on an Eksigent UPLC system. Separated peptides were analyzed by Orbitrap Velos Mass Spectrometers (ThermoFisher Scientific). A data‐dependent procedure that alternated between one full mass scan followed by the top 20 most intense precursor ions was applied with 90 s dynamic exclusion. Intact peptides were detected with a resolution of 30,000 at 400 m/z, and ion fragments were detected at 35%.

#### 2.3.5 Database search

Database search was performed with Maxquant search engine (v1.6.6.0) against the mouse proteome downloaded from UniProt. Trypsin was specified as cleavage enzyme allowing a maximum of 2 missing cleavages. Cysteine carbamidomethylation was set as a fixed modification. While methionine oxidation, N‐terminal acetylation of proteins, and lysine β‐hydroxybutyrylation were set as variable modifications. The false discovery rate (FDR) was set to 1% for peptide, protein, and modification site. Peptides identified from reverse or contaminant protein sequences, as well as peptides with a score below 40 or site localization probability below 0.75 were removed.

#### 2.3.6 Bioinformatics analysis

The Gene Ontology (GO) annotation proteome was obtained from the UniProt‐GOA database (http://www.ebi.ac.uk/GOA/). Proteins were annotated based on biological processes and molecular functions. Proteins were also annotated using the Kyoto Encyclopedia of Genes and Genomes (KEGG) database. KEGG enrichment analysis was carried out, with a *p* value <0.05 considered to be statistically significant.

### 2.4 Na‐β‐OHB treatment

Sodium β‐hydroxybutyrate (Na‐β‐OHB) was purchased from Sigma‐Aldrich (298360, USA). HT22 cells were treated with 10 mM Na‐β‐OHB or PBS for 24 h before further analysis.

### 2.5 LC–MS to study metabolite abundance

Treated HT22 cells were washed with pre‐cold PBS and quenched by ice‐cold 80% methanol solution. For mouse brain samples, brain tissues quenched by ice‐cold 80% methanol solution with homogenate. The quenched mixture was vortexed for 20 s to totally release the metabolites. After centrifuged at 12,000 ***g*** for 30 min at 4°C, the supernatant was collected and dried by a vacuum centrifugal concentrator at 4°C. The dried supernatants were dissolved in 50% acetonitrile solution and prepared for subsequent LC–MS analysis. LC–MS was performed as described previously to determine metabolite abundance (Zhang et al., 2022).

### 2.6 ATP measurement

ATP in treated HT22 cells or mouse brains was determined with the ATP Assay Kit (S0026, Beyotime, China), following the manufacturer's instructions.

### 2.7 α‐KGDH and MDH activity assays

Treated HT22 cells were used for α‐KGDH and MDH activity measurement with α‐Ketoglutarate Dehydrogenase Activity Assay Kit (ab185440, Abcam, UK) and NAD‐Malate Dehydrogenase Activity Assay Kit (E‐BC‐K561‐M, Elabscience, China), respectively.

### 2.8 CS activity assay

Homogenized mouse brain samples or treated HT22 cells were used for CS activity measurement with a CS activity assay kit (E‐BC‐K178‐M, Elabscience, China), following the manufacturer's instructions.

### 2.9 Western blot

Mouse brain tissues or treated cells were harvested and lysed with Pierce™ IP Lysis Buffer (87787, Thermo Scientific, USA) containing protease inhibitors (04693132001, Roche, Switzerland). Equal amounts of protein lysates were run on 10% SDS–PAGE gels (PN203, NCM, China). Separated proteins on gels were transferred to PVDF membranes. After blocking with 5% skim milk at room temperature for 1 h, membranes were incubated with primary antibodies at 4°C overnight. Membranes were then washed with TBS‐Tween20 for three times, incubated with appropriate secondary antibodies at room temperature for 1 h, and washed with TBS‐Tween20 for three times again. Protein signals were developed with NcmECL Ultra (P10300A, NCM, China) and detected using Azure C300 (Azure, USA). The quantitation of protein bands was conducted with ImageJ.

In some experiments, protein lysates were first incubated with indicated antibodies or IgG (30000‐0‐AP, Proteintech, China) overnight at 4°C, and then added with Protein A/G magnetic beads (B23201, Bimake, USA) for additional 1 h incubation at 4°C. The beads were washed with NP‐40 buffer. Immunoprecipitated proteins were then subjected to Western blot with indicated antibodies. For overexpressed Flag‐tagged proteins, protein lysates were incubated with anti‐Flag magnetic beads (A36797, Thermo Scientific, USA) overnight at 4°C. The beads were washed with NP‐40 buffer. Immunoprecipitated proteins were then subjected to Western blot with indicated antibodies.

Antibodies used in this study include: anti‐Kbhb Rabbit pAb (PTM‐1201, PTM Biolabs Inc., China, 1:1000); anti‐CS (67784‐1‐Ig, Proteintech, China, 1:1000); anti‐SUCLG1 (14923‐1‐AP, Proteintech, China, 1:1000); anti‐α‐Tubulin (66031‐1‐Ig, Proteintech, China, 1:100000); anti‐Flag (66008‐4‐Ig, Proteintech, China, 1:5000); anti‐mouse secondary antibody (G‐21040, Invitrogen, USA, 1:10000); and anti‐rabbit secondary antibody (G‐21234, Invitrogen, USA, 1:10000).

### 2.10 Lentivirus packaging and infection

293T cells were transfected with lentiviral vectors (LV) containing CS‐Flag‐puro, CS‐K52R‐Flag‐puro, CS‐K393R‐Flag‐puro, SUCLG1‐Flag‐puro or SUCLG1‐K81R‐Flag‐puro along with pMDL, pVSVG, and pREV, using Hieff Trans™ Liposomal transfection reagent (40802ES08, Yeasen, China) and following the manufacturer's protocol. Transfected cells were cultured for 48 h, and the media were collected and filtered with a 0.45 μm sterile filter unit (SLHVR33RB, Millipore, USA) to remove cellular debris.

HT22 cells grown to 40% confluency were infected with lentiviruses in the presence of 5 μg/mL Polybrenewere (H9268, Sigma‐Aldrich, USA). After 24 h, the media was replaced with fresh DMEM media, and cells were incubated for additional 48 h.

### 2.11 Ketogenic diet treatment

Six‐month‐old APP/PS1 male mice were fed with either control diet (XTKDCON, Xietong Pharmaceutical Bio‐engineering, China) or ketogenic diet (XTKD01, Xietong Pharmaceutical Bio‐engineering, China) ad libitum for 3 months. Following the KD treatment, 9‐month‐old APP/PS1 mice were subjected to cardiac perfusion. Brain tissues were subsequently used for metabolite detection and immunofluorescence staining.

### 2.12 Measurement of β‐OHB in plasma and liver

Blood samples were taken from tail vein of treated mice. Plasma was separated by centrifugation at 1500 *g* for 15 min at 4°C and frozen at −80°C until thawed for assay. The liver tissues were homogenized and sonicated 3 times on ice with a high‐intensity ultrasonic processor. Debris were removed by centrifugation at 12,000 ***g*** at 4°C for 10 min. The supernatants were collected for β‐OHB level detection. β‐OHB levels in Plasma and liver were measured using the beta‐Hydroxybutyrate Assay Kit (ab83390, Abcam, UK).

### 2.13 Immunofluorescence staining

Treated mice were anesthetized with sodium pentobarbital (60 mg/kg) and perfused intracardially with physiological saline. Brains were carefully extracted and fixed in 4% PFA overnight. Fixed tissues were dehydrated with 30% sucrose at 4°C. Sections (20 μm) of mouse brains were cut using a cryostat (CM3050S, Leica). After sectioning, brain slices were incubated with blocking buffer (0.3% Triton‐X‐100, and 2% BSA in PBS) for 1.5 h at room temperature. Slices were further incubated with antibodies against Aβ (GB111197, Servicebio, China, 1:200), Iba1 (019–19,741, Wako, Japan, 1:250; or 234,308, SYSY, Germany,1:500), GFAP (#3670, CST, USA, 1:200), NeuN (#3278580, Merk, USA, 1:50), Axl (13196‐1‐AP, Proteintech, China, 1:20), Clec7a (50233‐R010, SinoBiological, China, 1:20), CD68 (MCA1957GA, BIO‐RED, USA, 1:1000), or Kbhb (PTM‐1201, PTM Biolabs Inc., China, 1:50), and then with appropriate fluorescence‐labeled secondary antibodies (GB21303, Servicebio, China, 1:300; GB25301, Servicebio, China, 1:400; or 33412ES60, Yeasen, China, 1:200). Slices were covered on Anti‐fade Mounting Medium with DAPI (H‐1200, Vector, USA). Images were acquired with a laser confocal microscope (Nikon, Japan).

### 2.14 Quantification and statistical analysis

The numbers of mice in each group and experimental repeats are specified in the corresponding figure legends. All data were analyzed using GraphPad Prism 9 software and presented as means ± SEM. Unpaired two‐tailed Student's *t* test was used for two group comparisons. Statistical significance was defined as *p* < 0.05.

## 3 RESULTS

### 3.1 Identifying lysine β‐hydroxybutyrylation (Kbhb)‐modified proteins in APP/PS1 mice

**FIGURE 1:**
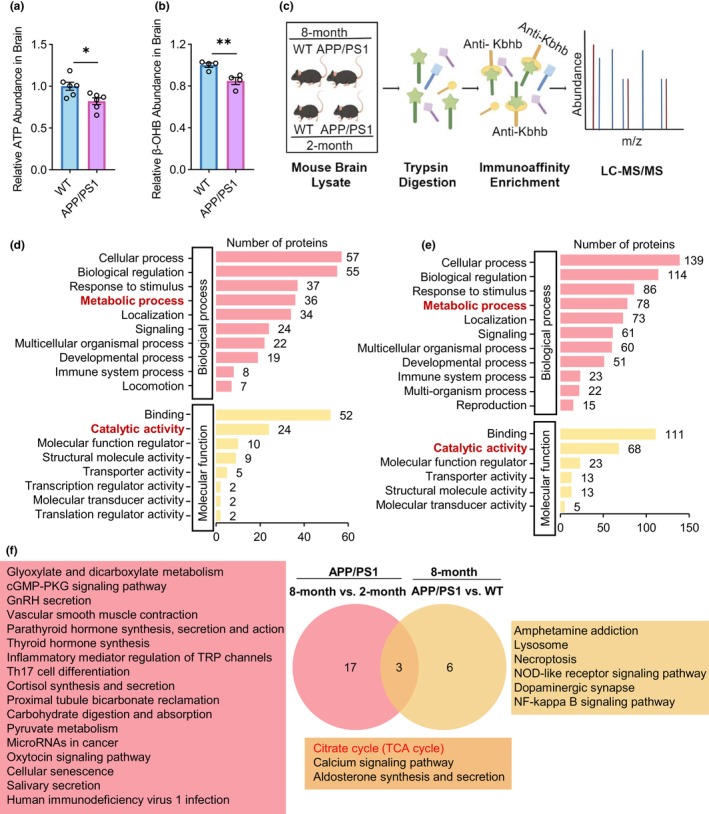
Characterizing lysine β‐hydroxybutyrylation during disease progression and onset in APP/PS1 mouse brain tissues. (a) Comparison of ATP abundance in the brain of 8‐month‐old APP/PS1 and WT mice. *n* = 6 per group. (b) Comparison of β‐OHB abundance in the brain of 8‐month‐old APP/PS1 and WT mice. *n* = 4 per group. (c) Scheme of lysine β‐hydroxybutyrylation (Kbhb) modified protein identification procedure (by Figdraw). LC, liquid chromatography; MS, mass spectrometry. (d) Gene Ontology (GO) classifications of proteins with downregulated Kbhb modifications in 8‐month‐old APP/PS1 versus 2‐month‐old APP/PS1 mice based on biological processes (pink) and molecular functions (yellow). (e) GO classifications of proteins with downregulated Kbhb modifications in 8‐month‐old APP/PS1 versus 8‐month‐old WT mice based on biological processes (pink) and molecular functions (yellow). (f) Comparison of enriched KEGG pathways for proteins with downregulated Kbhb modifications in 8‐month‐old APP/PS1 versus 2‐month‐old APP/PS1 mice (pink) and for those in 8‐month‐old APP/PS1 versus 8‐month‐old WT mice (yellow). The obtained data were subjected to Unpaired Student's *t* test analysis and presented as means ± SEM. *p < 0.05, **p < 0.01.

The presence of an energy deficit in the brain is considered one of the pathological features of AD. In this study, we investigated the abundance of ATP in the brains of 8‐month‐old APP/PS1 mice and WT mice. Our results showed a significant decrease in ATP levels in the brain of APP/PS1 mice compared to WT controls (Figure 1a). This finding suggests that APP/PS1 mice could mimic the energy deficit observed in AD. β‐OHB as the inducer of protein Kbhb modification has been reported decreased in AD brain (Shippy et al., 2020). Herein, we measured β‐OHB abundance in the brains of APP/PS1 and their littermate WT mice at 8 months old and found that β‐OHB levels were decreased in the brains of APP/PS1 mice (Figure 1b). Given that Kbhb plays an important role in regulating protein function (Cornuti et al., 2023; Koronowski et al., 2021), we hypothesize that decreased β‐OHB levels lead to dysregulation of protein Kbhb, which may be one of the mechanisms underlying AD pathogenesis. To investigate the role of protein Kbhb in AD, we conducted 4D label‐free Kbhb quantitative proteomics in four groups of mice (Figure 1c), which include 8‐month‐old APP/PS1 mice that exhibit marked Aβ plaque burdens and cognitive impairment, 2‐month‐old APP/PS1 mice that have little Aβ plaques and no cognitive impairment (Gengler et al., 2010; Radde et al., 2006; Serneels et al., 2009), and 2‐month‐old and 8‐month‐old littermate WT mice.

We compared Kbhb‐modified protein sites between different mouse groups. 132 sites on 129 proteins were identified to be differentially β‐hydroxybutylated in the comparison between 8‐month‐old APP/PS1 and 2‐month‐old APP/PS1 mice, representing Kbhb modification changes during disease progression. 194 sites on 184 proteins were identified to be differentially β‐hydroxybutylated in the comparison between 8‐month‐old APP/PS1 and 8‐month‐old WT mice, representing Kbhb modification changes during disease onset. 234 sites on 225 proteins were identified to be differentially β‐hydroxybutylated in the comparison between 2‐month‐old APP/PS1 and 2‐month‐old WT mice, representing Kbhb modification changes associated with the asymptomatic period of disease. Lastly, 187 sites on 187 proteins were identified to be differentially β‐hydroxybutylated in the comparison between 2‐month‐old WT and 8‐month‐old WT mice, representing Kbhb modification changes associated with the normal growth process (Table S1a–e).

Since β‐OHB abundance is decreased in the brain of AD patients and animal models, we focused on proteins with downregulated Kbhb modifications during disease progression (8‐month‐old APP/PS1 vs. 2‐month‐old APP/PS1) and disease onset (8‐month‐old APP/PS1 vs. 8‐month‐old WT). GO functional classifications revealed that proteins with downregulated Kbhb modifications during disease progression (Figure 1d) and onset (Figure 1e) were similarly involved in biological processes such as cellular process, biological regulation, response to stimulus, metabolic process, and localization, and similarly involved in molecular functions such as binding, catalytic activity, molecular function regulation, and transporter activity.

We also performed KEGG enrichment analysis and identified 20 enriched pathways for proteins with downregulated Kbhb modifications during disease progression and nine enriched pathways for proteins with downregulated Kbhb modifications during disease onset (Figure 1f). Among them, we found that three pathways were shared during disease progression and onset, including citrate cycle (TCA cycle), calcium signaling pathway, and aldosterone synthesis and secretion (Figure 1f). While the three pathways were not found in enriched pathways for proteins with downregulated Kbhb modifications during the asymptomatic period or normal growth process (Figure S1).

### 3.2 β‐OHB enhances SUCLG1 and CS activity by inducing Kbhb modification

**FIGURE 2:**
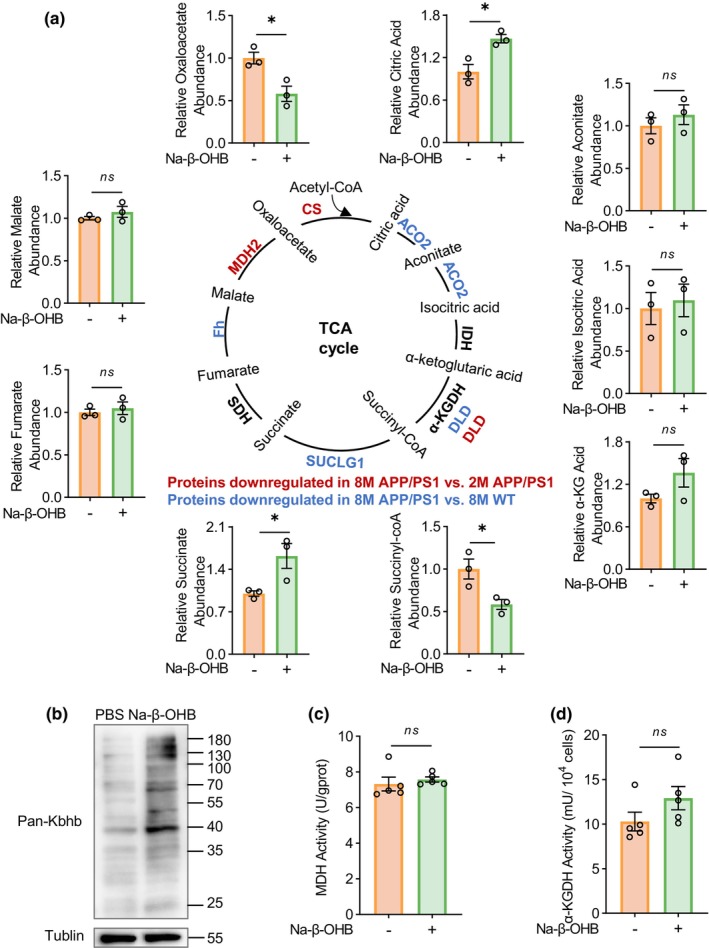
Identifying TCA cycle‐associated enzymes whose Kbhb modification affects their activity. (a) TCA cycle‐associated enzymes with downregulated Kbhb modifications in 8‐month‐old APP/PS1 versus 2‐month‐old APP/PS1 mice were highlighted in red. Those with downregulated Kbhb modifications in 8‐month‐old APP/PS1 versus 8‐month‐old WT mice were highlighted in blue. The relative abundance of metabolites in TCA cycle in HT22 cells treated with or without 10 mM Na‐β‐OHB for 24 h were measured by LC–MS for comparisons. *n* = 3 per group (b) Global protein Kbhb modifications in HT22 cells treated with Na‐β‐OHB were checked by Western blot using an anti‐pan‐Kbhb antibody. (c, d) The MDH activity (c) and the α‐KGDH activity (d) in HT22 cells treated with or without 10 mM Na‐β‐OHB for 24 h were measured for comparisons. *n* = 5 per group. The obtained data were subjected to Unpaired Student's *t* test analysis and presented as means ± SEM. *p < 0.05, ns, no significant differences (*p* > 0.05).

β‐OHB is a major component of ketone bodies that serve as alternative energy sources for the brain. Since glucose hypometabolism in the brain is an important pathological feature in AD, we focused on the TCA cycle that controls ATP production and was identified to be associated with altered protein Kbhb modifications during disease progression and onset in the current study. Figure 2a illustrates the TCA cycle‐associated proteins whose Kbhb modifications were downregulated during disease progression and onset. Proteins with downregulated Kbhb modifications during disease progression included citrate synthase (CS), malate dehydrogenase (MDH2), and dihydrolipoyl dehydrogenase (DLD). Proteins with downregulated Kbhb modifications during disease onset included aconitate hydratase (ACO2), dihydrolipoyl dehydrogenase (DLD), succinate‐CoA ligase subunit alpha (SUCLG1), and fumarate hydratase (Fh).

To explore which protein Kbhb modifications are critically involved in glucose metabolism in the TCA cycle, we treated HT22 cells with Na‐β‐OHB and analyzed metabolite levels by LC/MS. Na‐β‐OHB treatment dramatically increased protein Kbhb levels (Figure 2b). LC–MS analysis showed that oxaloacetate levels were significantly decreased and citric acid levels were significantly increased upon Na‐β‐OHB treatment (Figure 2a). Since CS catabolizes oxaloacetate to citric acid, these results suggest that Kbhb modification of CS promotes its enzymatic activity and this is important for the TCA cycle. Similarly, SUCLG1 catabolizes succinyl‐CoA to succinate and because succinyl‐CoA levels were significantly decreased and succinate levels were significantly increased upon Na‐β‐OHB treatment (Figure 2a), these results indicate that Kbhb modification of SUCLG1 promotes its enzymatic activity and this is also important for the TCA cycle. However, for ACO2 that catabolizes citric acid to aconitate and then to isocitric acid, isocitrate dehydrogenase (IDH) that catabolizes isocitric acid to α‐ketoglutaric acid, α‐ketoglutarate dehydrogenase (α‐KGDH) that catabolize α‐ketoglutaric acid to succinyl‐CoA, succinate dehydrogenase (SDH) that catabolizes succinate to fumarate, fumarate dehydrogenase (Fh) that catabolizes fumarate to malate, and malate dehydrogenase 2 (MDH2) that catabolizes malate to oxaloacetate, their substrate levels and/or product levels were not significantly changed upon Na‐β‐OHB treatment (Figure 2a), implicating that Kbhb modifications of these proteins may not markedly affect the TCA cycle. Furthermore, we measured MDH and α‐KGDH activities and consistently found them not affected by Na‐β‐OHB treatment (Figure 2c,d).

**FIGURE 3:**
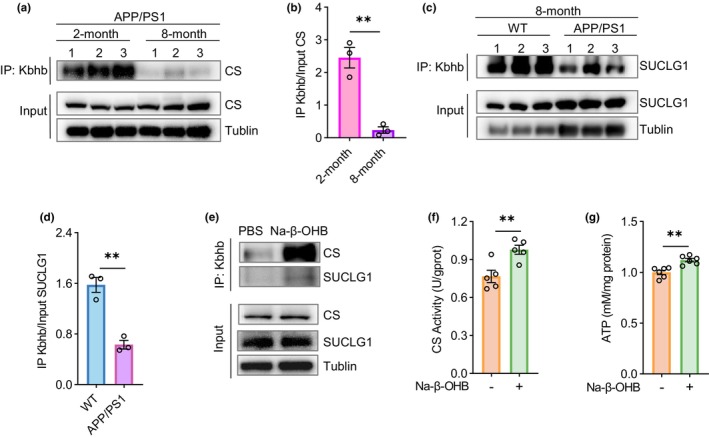
Kbhb modifications of CS and SUCLG1 affect their enzymatic activities. (a) Equal amounts of protein lysates from mouse brain tissues were subjected to immunoprecipitation (IP) with anti‐pan‐Kbhb antibody and Western blot with anti‐CS antibody. (b) Comparison of CS Kbhb modification levels in (a). *n* = 3 per group. (c) Equal amounts of protein lysates from mouse brain tissues were subjected to IP with an anti‐pan‐Kbhb antibody and Western blot with an anti‐SUCLG1 antibody. (d) Comparison of SUCLG1 Kbhb modification levels in (c). *n* = 3 per group. (e) HT22 cells were treated with 10 mM Na‐β‐OHB for 24 h. Cell lysates were subjected to IP with an anti‐pan‐Kbhb antibody and Western blot with anti‐CS or anti‐SUCLG1 antibodies. (f, g) HT22 cells were treated with or without 10 mM Na‐β‐OHB for 24 h. The CS activity (f) and ATP (g) levels were measured for comparisons. For CS activity, *n* = 5 per group; for ATP levels, *n* = 6 per group. The obtained data were subjected to Unpaired Student's *t* test analysis and presented as means ± SEM. **p < 0.01.

Therefore, we focused on functional studies on CS and SUCLG1. We performed immunoprecipitation (IP) with an anti‐pan‐Kbhb antibody using mouse brain samples and then immunoblotted with antibodies against CS and SUCLG1. The results showed that consistent with the proteomic findings, CS Kbhb modification (Figure 3a,b) was significantly decreased in 8‐month‐old APP/PS1 mice compared to 2‐month‐old APP/PS1 mice and SUCLG1 Kbhb modification (Figure 3c,d) was significantly decreased in 8‐month‐old APP/PS1 mice compared to 8‐month‐old WT mice.

On the contrary, we found that Na‐β‐OHB treatment significantly increased CS and SUCLG1 Kbhb modifications in HT22 cells (Figure 3e). Na‐β‐OHB treatment also significantly enhanced the CS enzymatic activity (Figure 3f) and increased ATP content (Figure 3g). These findings collectively support the notion that Na‐β‐OHB treatment enhances ATP production through promoting Kbhb modifications of CS and SUCLG1 and thereby their enzymatic activities.

### 3.3 Kbhb modifications of CS K393 and SUCLG1 K81 are important for their enzymatic activities

**FIGURE 4:**
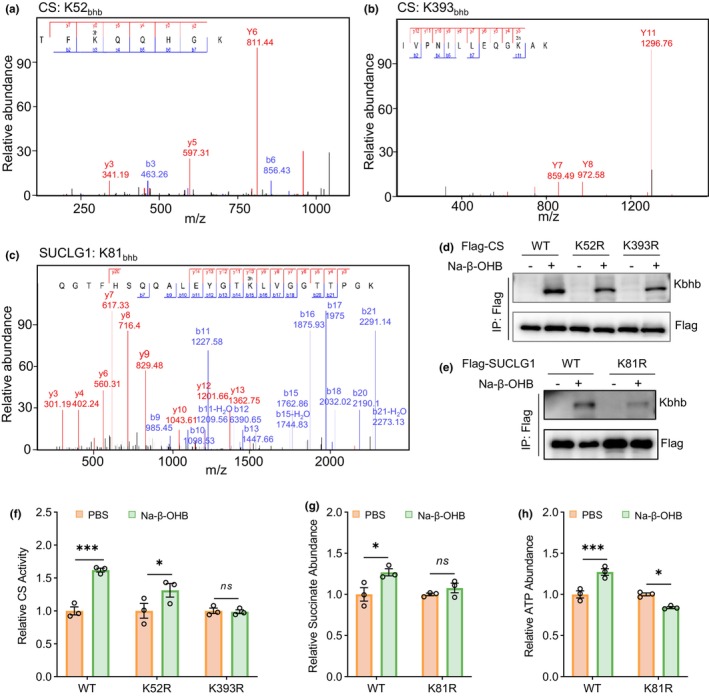
K393 Kbhb modification in CS and K81 Kbhb modification in SUCLG1 are crucial for respective enzymatic activities. (a, b) MS spectra of CS K52 (a) and K393 (b) Kbhb modifications. (c) MS spectra of SUCLG1 K81 Kbhb modification. (d) HT22 cells were infected with lentiviruses expressing flag‐tagged WT CS, CS K52R mutant, or CS K393R mutant. After treatment with 10 mM Na‐β‐OHB for 24 h, CS Kbhb modifications were detected by immunoprecipitation (IP) with an anti‐flag antibody first and then Western blotting with an anti‐pan‐Kbhb antibody. (e) HT22 cells were infected with lentiviruses expressing flag‐tagged WT SUCLG1 or SUCLG1 K81R mutant. After Na‐β‐OHB treatment, SUCLG1 Kbhb modifications were analyzed. (f) HT22 cells were infected with lentiviruses expressing different CS variants and treated with Na‐β‐OHB. The CS activity was assayed for comparison. *n* = 3 per group. (g, h) HT22 cells were infected with lentiviruses expressing different SUCLG1 variants and treated with Na‐β‐OHB. The succinate abundance (g) and ATP levels (h) were measured for comparisons. *n* = 3 per group. The obtained data were subjected to Unpaired Student's *t* test analysis and presented as means ± SEM. *p < 0.05, ***p < 0.001; ns, no significant differences (*p* > 0.05).

Proteomic analysis showed Kbhb modifications of CS at lysine (K) residues 52 and 393 (Figure 4a,b), and Kbhb modification of SUCLG1 at lysine residue 81 (Figure 4c). To determine whether Kbhb modifications of these sites are important for their enzymatic activities, we infected HT22 cells with lentiviruses expressing wild type (WT) CS, CS K52R mutant or CS K393R mutant and then treated with Na‐β‐OHB. We found that both K52R and K393R mutations markedly decreased CS Kbhb modifications (Figure 4d). While Na‐β‐OHB treatment significantly promoted CS enzymatic activity in cells expressing WT CS and CS K52R mutant, but not in cells expressing CS K393R mutant (Figure 4f). These results suggest that Kbhb modification of CS K393 site is crucial for its enzymatic activity.

In HT22 cells infected with lentiviruses expressing WT SUCLG1 or SUCLG1 K81R mutant, we found that K81R mutation dramatically decreased SUCLG1 Kbhb modification (Figure 4e). In addition, overexpression of SUCLG1 K81R mutant could not increase succinate levels as overexpression of WT SUCLG1 did upon Na‐β‐OHB treatment (Figure 4g). Moreover, Na‐β‐OHB treatment increased ATP abundance in cells overexpressing WT SUCLG1 but reduced ATP abundance in cells overexpressing SUCLG1 K81R mutant (Figure 4h). These results indicate that Kbhb modification of SUCLG1 K81 site is important for its enzymatic activity.

### 3.4 Ketogenic diet enhances ATP production in APP/PS1 mice by increasing CS and SUCLG1 Kbhb modifications and their enzymatic activities

**FIGURE 5:**
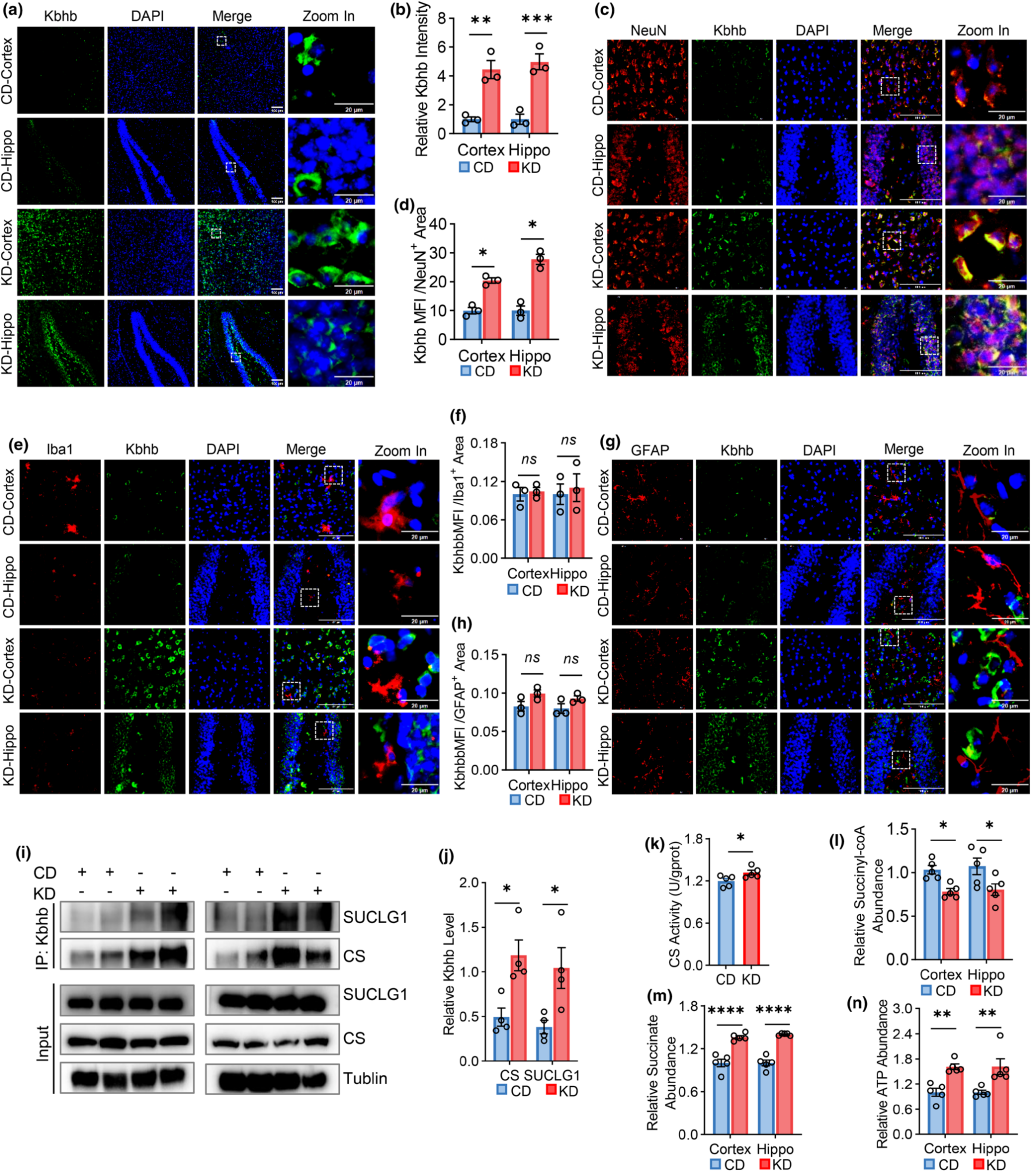
Ketogenic diet enhances ATP production in APP/PS1 mice by increasing CS and SUCLG1 Kbhb modifications and their enzymatic activities. (a) Representative images of immunofluorescence staining of global Kbhb modifications in the hippocampus and cortex of APP/PS1 mice fed with CD or KD. Green, pan‐Kbhb; blue, DAPI. Scale bars =100 μm or 20 μm. (b) Comparison of Kbhb intensities in (a). *n* = 3 per group. (c, e, g) Representative images of pan‐Kbhb co‐immunostained with NeuN (c), Iba1 (e), and GFAP (g) in the hippocampus and cortex of APP/PS1 mice fed with CD or KD. Red, NeuN (c), Iba1 (e), or GFAP (g); green, pan‐Kbhb; blue, DAPI. Scale bars = 100 μm or 20 μm. (d, f, h) Comparison of Kbhb intensities in NeuN‐ (d), Iba1‐ (f), or GFAP‐ (h) positive cells. *n* = 3 per group. (i) Kbhb levels of CS and SUCLG1 in the brain tissues of APP/PS1 mice fed with CD or KD were studied by IP with an anti‐pan‐Kbhb antibody and Western blot with indicated antibodies. (j) Comparison of CS and SUCLG1 Kbhb levels in (i). *n* = 4 per group. (k) Comparison of CS activity in the brain of APP/PS1 mice fed with CD or KD. *n* = 5 per group. (l–n) Comparisons of succinyl‐coA abundance (l), succinate abundance (m), and ATP levels (n) in the hippocampus and cortex of APP/PS1 mice fed with CD or KD. *n* = 5 per group. The obtained data were subjected to Unpaired Student's *t* test analysis and presented as means ± SEM. *p < 0.05; **p < 0.01; ***p < 0.001, ****p < 0.0001, ns, no significant differences (*p* > 0.05).

Ketogenic diet (KD) is recognized as an effective intervention for enhancing ketogenic metabolism and increasing β‐OHB level in vivo. We administered KD to 6‐month‐old APP/PS1 mice for 3 months. KD treatment significantly increased the abundance of β‐OHB in mouse liver, plasma, and brain (Figure S2a–c). Furthermore, KD treatment resulted in a notable increase in global protein Kbhb modification in both the hippocampus and cortex as demonstrated by immunofluorescence staining (Figure 5a,b). To identify the primary cell type responding to Kbhb, we conducted co‐immunostaining of pan‐Kbhb with markers of neurons (NeuN), microglia (Iba1), and astrocytes (GFAP). The results revealed that KD treatment significantly elevated Kbhb levels in neurons (Figure 5c,d), while showing minimal changes in microglia and astrocytes (Figure 5e–h). We further investigated and found that KD significantly increased Kbhb modification levels of CS and SUCLG1 in the brain of APP/PS1 mice (Figure 5i,j). Additionally, the activities of CS and SUCLG1 were found to be significantly increased in brain tissues of KD‐treated APP/PS1 mice, as assessed by CS enzyme activity assay (Figure 5k) and measurements of succinyl‐CoA and succinate (Figure 5l,m). Moreover, ATP production in the brain of APP/PS1 mice was also found to be increased following KD treatment (Figure 5n).

### 3.5 Ketogenic diet (KD) attenuates AD‐associated pathologies in APP/PS1 mice

**FIGURE 6:**
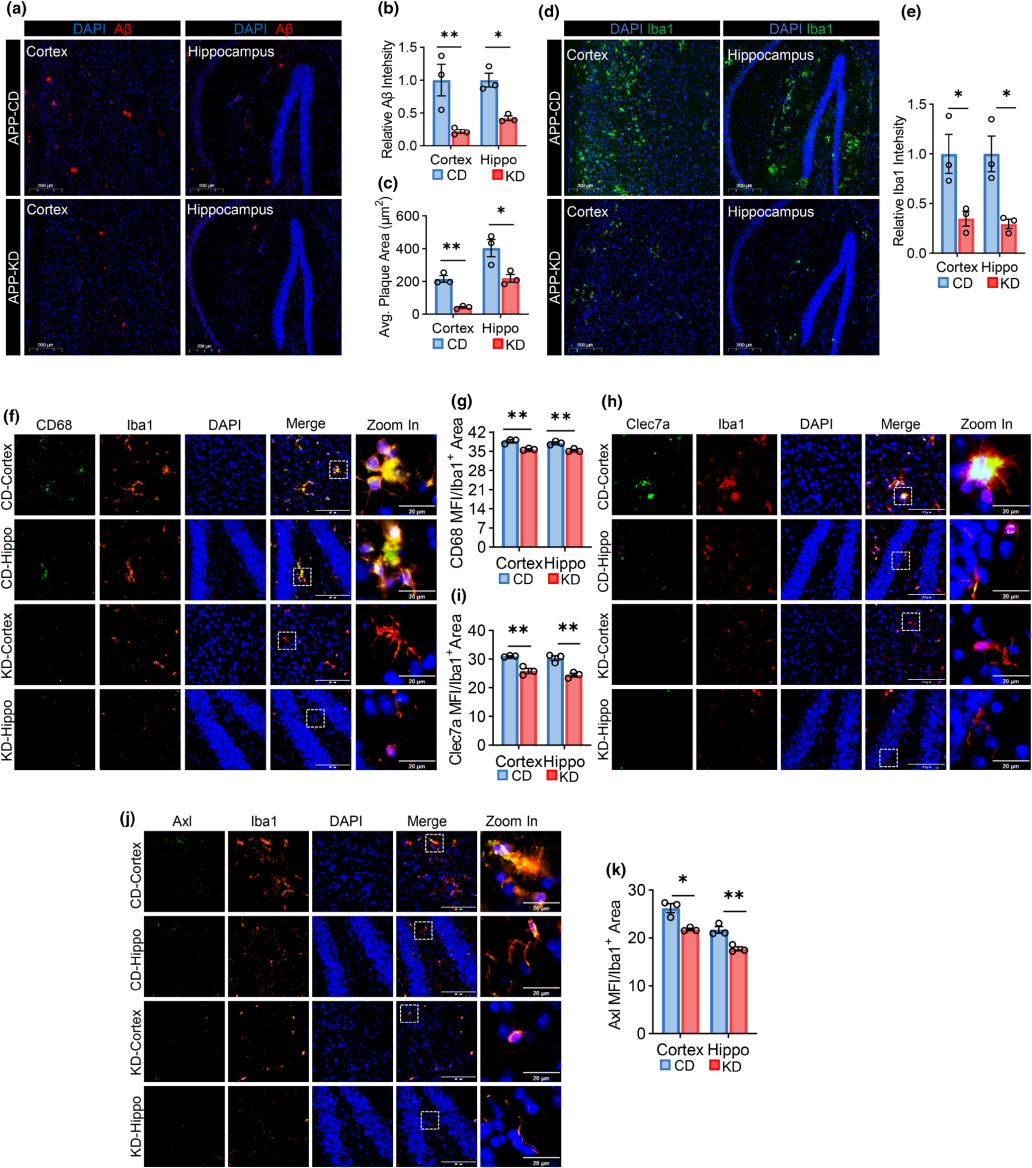
Ketogenic diet (KD) treatment attenuates APP/PS1 mouse pathologies. (a) Representative images of immunofluorescence staining of Aβ plaques in the hippocampus and cortex of APP/PS1 mice fed with CD or KD. Red, Aβ; blue, DAPI. Scale bars = 200 μm. (b, c) Comparison of Aβ intensity (b) and average size (c) in (a). *n* = 3 per group. (d) Representative images of immunofluorescence staining of Iba1 in the hippocampus and cortex of APP/PS1 mice fed with CD or KD. Green, Iba1; blue, DAPI. Scale bars = 200 μm. (e) Comparison of Iba1 intensity in (d). *n* = 3 per group. (f, h, j) Representative images of Iba1 co‐immunostained with CD68 (f), Clec7a (h), or Axl (j) in the hippocampus and cortex of APP/PS1 fed with CD or KD. Red, Iba1; green, CD68 (f), Clec7a (h), or Axl (j); blue, DAPI. Scale bars = 100 μm or 20 μm. (g, i, k) Comparisons of CD68 (g), Clec7a (i), or Axl (k) intensity in Iba1 positive cells. *n* = 3 per group. The obtained data were subjected to Unpaired Student's *t* test analysis and presented as means ± SEM. *p < 0.05; **p < 0.01.

Given that ketogenic diet induces protein Kbhb and enhances ATP production in the brains of APP/PS1 mice, we further investigated whether KD treatment ameliorated the pathology of APP/PS1 mice. The results showed that KD treatment led to a significant reduction in Aβ plaque burden (Figure 6a–c). Additionally, we observed a notable decrease in Iba1 intensity following KD treatment (Figure 6d,e), suggesting that KD ameliorates microgliosis. CD68 is a marker for activated pro‐inflammatory microglia (Wei & Li, 2022). Clec7a is a marker of disease‐associated microglia (DAMs) (Barclay et al., 2024). Axl is a marker of plaque‐associated microglia (PAMs) (Huang et al., 2021). We also assessed the intensities of these markers and found that all of them decreased in microglia following KD treatment (Figure 6f–k), further confirming that KD attenuates microglia overactivation in APP/PS1 mice.

## 4 DISCUSSION

β‐OHB is a main product of ketogenesis and serves as an alternative energy source during glucose deficiency (Newman & Verdin, 2014). β‐OHB can be converted into acetyl‐CoA through ketolysis for ATP production in the TCA cycle. More recently, β‐OHB has been found to regulate protein Kbhb modification, a type of post‐translational modification (Koronowski et al., 2021; Xie et al., 2016). Herein, we identified multiple proteins that are subjected to Kbhb modification in both WT and APP/PS1 mice, expanding the reservoir of proteins with Kbhb modification.

Although a few studies have revealed that protein Kbhb modification is involved in regulating gene expression, liver metabolism, cell growth arrest, and apoptosis, etc. (Koronowski et al., 2021; Li et al., 2022; Liu et al., 2019; Xie et al., 2016), whether and how protein Kbhb participates in AD remained unknown. In the present study, we found that accompanying decreased β‐OHB levels, proteins with decreased Kbhb modifications in the brain of APP/PS1 mice at pathological stages were enriched in the TCA cycle, implicating that altered protein Kbhb modifications during disease progression and onset have marked effects on the TCA cycle function. Indeed, Kbhb modifications of CS and SUCLG1, two important enzymes in the TCA cycle were decreased in APP/PS1 mice at pathological stages. While β‐OHB treatment significantly increased Kbhb modifications of CS and SUCLG1 and their enzymatic activities, leading to elevated ATP production. These findings for the first time demonstrate that in addition to being converted into acetyl‐CoA, β‐OHB regulates the TCA cycle through modifying Kbhb of TCA cycle‐associated enzymes.

Glucose hypometabolism is an important pathological feature and has been used as a biomarker for AD (Austad et al., 2022; Scheltens et al., 2016). Studies have shown a decrease of 21%–28% in glucose uptake and utilization in the brain of AD patients compared to normal controls, as observed through Positron Emission Tomography (PET) imaging using ^18^F‐fluorodeoxyglucose (FDG) (de Leon, Ferris, et al., 1983; de Leon, George, et al., 1983). Further investigations have revealed impairments in glucose metabolism including the TCA cycle in AD patients (Brooks et al., 2007; Bubber et al., 2005). Hence, the concept of energy therapy has been proposed for treating energy deficits in AD. One form of energy therapy is KD, which involves using ketone bodies as an alternative energy source to replenish energy in AD brain (Cunnane et al., 2011, 2020). Several studies have indicated that KD enhances cognition and memory in both AD patients and animal models. For example, supplementation of MCFA (both C8 and C10) for 1 month improved metabolism in AD patients (Croteau et al., 2018). β‐OHB treatment alleviated cognition and memory deficits, Aβ accumulation, microglia overactivation, and neuroinflammation in 5xFAD mice (Shippy et al., 2020; Wu et al., 2020). β‐OHB treatment also impeded the progression of AD‐related phenotypes in ApoE‐deficient mice (Krishnan et al., 2020) and increased intermediates of the TCA cycle in the hippocampus of 3XTg AD mice (Pawlosky et al., 2017). Consistent with previous studies, here we found that KD reduced Aβ plaque burden in APP/PS1 mice. Moreover, we found that KD attenuated microgliosis, as revealed by the decreased numbers of microglia and the reduced expression of CD68, Clec7a, and Axl in microglia. CD68^+^ microglia function in pro‐inflammatory response (Wei & Li, 2022), and Clec7a^+^ microglia are disease‐associated microglia (DAMs) (Barclay et al., 2024). A previous study also reported that KD treatment decreased the expression of Clec7a and CD68 in APP/PS1 mice (Di Lucente et al., 2024). Axl is crucial for microglia to detect and engulf Aβ plaques, and its expression is prominently upregulated in microglia stimulated by inflammatory environments (Fourgeaud et al., 2016) and in plaque‐associated microglia (PAMs) (Huang et al., 2021). The reduction of Axl^+^ microglia is consistent with the reduction of Aβ plaque burden upon KD treatment.

Another thing interesting is that we found that KD‐induced pan‐Kbhb primarily occurred in neurons rather than microglia or astrocytes. This finding implicates that KD treatment mainly promotes the TCA cycle and ATP production in neurons. The decreased microgliosis upon KD treatment may be attributed to improved neuronal healthy, which reduces the stimulation of microgliosis. Whether and how different cell types responds specifically to protein Kbhb deserves further scrutiny.

In conclusion, our study identifies a series of proteins that undergo Kbhb modification and are associated with the progression and onset of AD. We demonstrate that the TCA cycle is mostly affected by β‐OHB reduction, which decreases Kbhb modifications and enzymatic activities of TCA cycle‐associated enzymes, especially CS and SUCLG1 in AD model mice at pathological stages. Moreover, we show that KD increases β‐OHB abundance, promotes Kbhb modifications of CS and SUCLG1 and their enzymatic activities, and elevates ATP production in the brain of APP/PS1 mice. Together, our findings not only identify a novel role of protein Kbhb modification in regulating TCA cycle, but also reveal a new mechanism underlying the beneficial effect of ketogenic diet on AD intervention.

## AUTHOR CONTRIBUTIONS

Y.W.Z. and Z.W. conceived the study, designed the experiments, and supervised the study. W.H., B.Z., W.Z., W.Z., J.H., X.Q., L.Z., X.W., Y.W., H.L., Y.X., Y.G., W.Z., and Q.H. performed experiments. W.H., B.Z., Y.W.Z., and Z.W. analyzed data. W.H. and B.Z. wrote the draft. Y.W.Z. and Z.W. reviewed and edited the manuscript. All authors have read and approved the article.

## CONFLICT OF INTEREST STATEMENT

The authors declare no conflict of interest.

## Supporting information


Figures S1–S2.

Table S1.

## Data Availability

All data generated or analyzed during this study were included in the manuscript.
