# Supplementary material for: Ketogenic β‐hydroxybutyrate regulates β‐hydroxybutyrylation of TCA cycle‐associated enzymes and attenuates disease‐associated pathologies in Alzheimer's mice
Source: Aging Cell. 2024 Oct 16;24(1):e14368. doi: 10.1111/acel.14368 (PMC11709107; doi:10.1111/acel.14368)
Supplement: Supplementary file 1 — Figures S1–S2. [file ACEL-24-e14368-s002.docx]

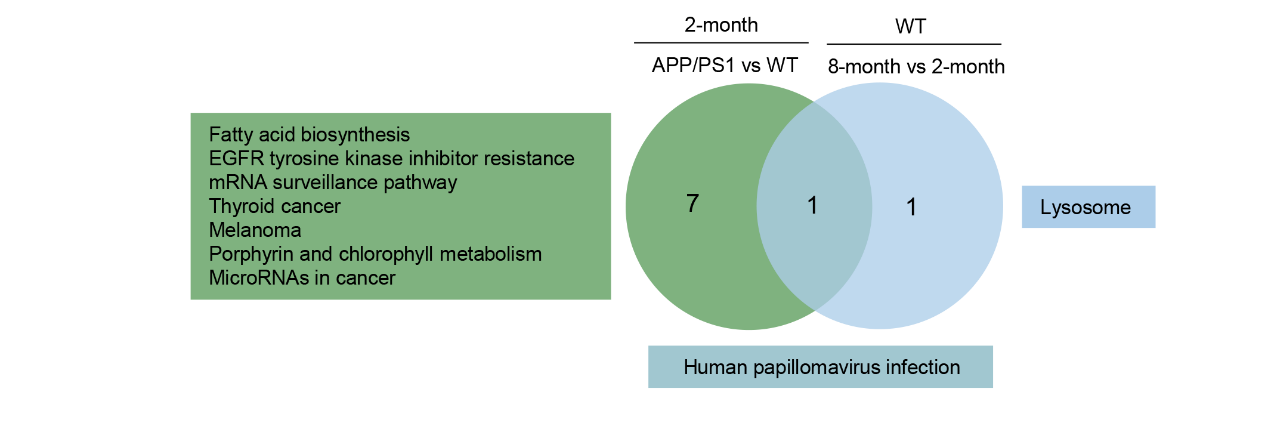


**Figure S1 Comparison of enriched KEGG pathways for proteins with downregulated Kbhb modifications in 2-month-old APP/PS1 vs 2-month-old WT mice (green) and for those in 8-month-old WT vs 2-month-old WT mice (blue).**


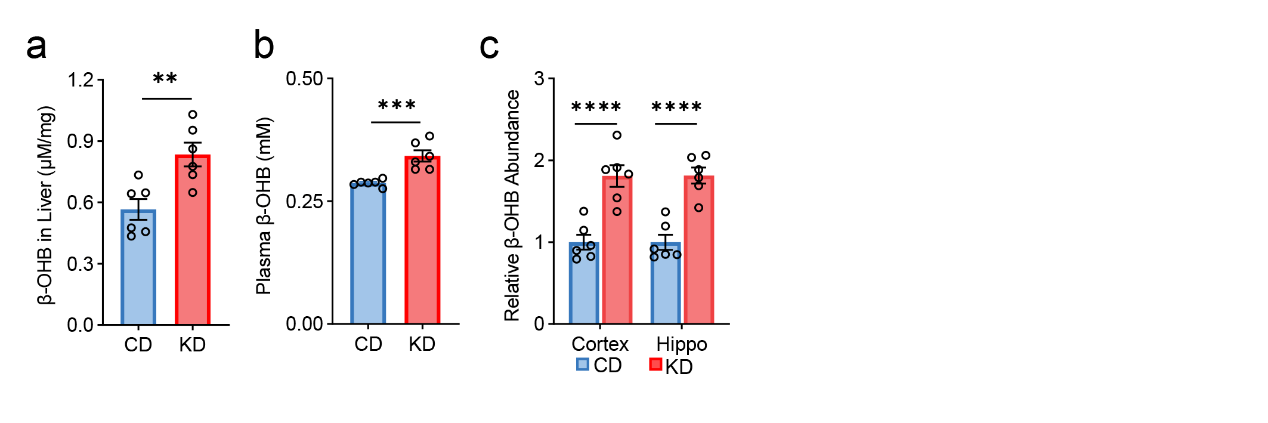


**Figure S2 Ketogenic diet increases β-OHB abundance.** (a-c) Comparison of β-OHB abundance in liver (a), plasma (b), and brain (c) of APP/PS1 mice fed with control diet (CD) or ketogenic diet (KD). n=6 per group. The obtained data were subjected to Unpaired Student’s t test analysis and presented as means ± SEM. ***p*<0.01, ****p*<0.001, *****p*<0.0001.
